# On the Unwholesomeness of Tea. From the French, of Mr. C. L. Cadet

**Published:** 1810-09

**Authors:** 


### *On the Unwhlesomeness of Tea. From the French, of* Mr. C. L. CADET.

TEA, before it is dried, is of a more or less deep fine green colour: its taste is bitter and stiptic. The teas that are sold, vary in their appearance, as some have passed through boiling water, and others are only dried. In general, imperial tea is deep green, green tea is puce green, hyson is blueish green, bohea is yellowish green, peko is almost black, gunpowder tea is greyish green, souchong is reddish. The odour of tea is equally various, and does not belong to the plant itself, being communicated to it by other aromatic plants, as chloranthus, olea fragrans, commelina, sesaaqua, Arabian jasmine, and curcuma; besides Florentine orris, which the retailers put at the bottom of their cannisters.

The

The infusion of tea, made at 70° or 80°, which is the usual heat, does not redden infusion of litmus. Mineral acids enliven the colour when they are dilute, and destroy it when they are concentrated. Alkalies turn it brown. It precipitates sulphate of iron black, and it coagulates a solution of glue.

Decoction of tea has the same properties, and also lets fall mucilage, on alkohol being added to it. When the decoction is very strong, it dyes woollen, by the help of a mordant, of a good nankeen colour.

A tincture of tea made with alkohol, yields ink with sulphate of iron, and contains a large quantity of resin mixed with extractive matter. Half an ounce of tea yielded a drachm and a half of resinous extract. This tincture dyes silk of a fawn colour.

Tea appears to contain extractive matter, mucilage, a large proportion of resin, gallic acid, and tannin. The two last principles explain the febrifuge quality assigned to tea by some medical men. It is not necessary to mention the fixed principles of tea. When, however, the leaves of tea, which had been infused, were dried and burned at the flame of a candle, the edge of the flame was tinged of a green colour; nevertheless, the most accurate trials could not discover the least trace of copper, either in the leaves or in their ashes: there were only found charcoal, iron, and muriate of alumine, but no potash, so that if any copper be present, it must be too small a proportion to be hurtful.

Superfine hyson contains the greatest quantity of gallic acid, then gunpowder tea, pouchong, souchong, imperial, hysonskin, green, tokai, peko, and lastly, bohea.

Superfine hyson also contains most tannin, then gunpowder tea, imperial, and souchong; the other sorts do not contain any.

Superfine hyson is most abundant in resin, then imperial, gunpowder tea, souchong, hysonsekin, green, tokai, pouchong, peko, and lastly bohea.

Peko contains most mucilage, then pouchong, hysonsekin, green, lastly tokai and the other kinds.

Superfine hyson contains most extractive matter, then imperial, gunpowder tea, souchong, hysonsekin, green, tokai, pouchong, peko, and lastly bohea.

Hence it appears that the teas which are most carefully prepared, contain most of the astringent and resinous principles; and as these teas are carefully roasted, it is probable that the roasting developes these principles. Bohea, and and peko, which are gathered in May, and have probably been infused in water before they were dried and rolled, contain very little astringent matter; but the peko contains so much mucilage, that its decoction draws out in threads, like that of linseed.

Again, for a styptic and astringent drink, hyson or gunpowder tea must be used; for a slight tonic without any astringency, green tea, or tokai; for an emollient and detersive, bohea or peko.

The leaves of tea when fresh gathered, are of a very disagreeable bitterness; and have so strong an action upon the nervous system, that they even occasion delirium; but the preparation given to it, and the length of time it is kept before it is used, diminish these deleterious effects, although perhaps they do not entirely remove them.

